# The critical roles of activated stellate cells-mediated paracrine signaling, metabolism and onco-immunology in pancreatic ductal adenocarcinoma

**DOI:** 10.1186/s12943-018-0815-z

**Published:** 2018-02-19

**Authors:** Yaojie Fu, Shanshan Liu, Shan Zeng, Hong Shen

**Affiliations:** 1 Department of Oncology, Xiangya Hospital, Central South University, Changsha, Hunan 410008 China; 2 Institute of Medical Sciences, Xiangya Hospital, Central South University, Changsha, Hunan 410008 China; 3 Key Laboratory for Molecular Radiation Oncology of Hunan Province, Xiangya Hospital, Central South University, Changsha, Hunan 410008 China

**Keywords:** Pancreatic stellate cells, PDAC, Metabolic reprogramming, Immune evasion, Drug resistance

## Abstract

Pancreatic ductal adenocarcinoma (PDAC) is one of the most lethal malignant diseases worldwide. It is refractory to conventional treatments, and consequently has a documented 5-year survival rate as low as 7%. Increasing evidence indicates that activated pancreatic stellate cells (PSCs), one of the stromal components in tumor microenvironment (TME), play a crucial part in the desmoplasia, carcinogenesis, aggressiveness, metastasis associated with PDAC. Despite the current understanding of PSCs as a “partner in crime” to PDAC, detailed regulatory roles of PSCs and related microenvironment remain obscure. In addition to multiple paracrine signaling pathways, recent research has confirmed that PSCs-mediated tumor microenvironment may influence behaviors of PDAC via diverse mechanisms, such as rewiring metabolic networks, suppressing immune responses. These new activities are closely linked with treatment and prognosis of PDAC. In this review, we discuss the recent advances regarding new functions of activated PSCs, including PSCs-cancer cells interaction, mechanisms involved in immunosuppressive regulation, and metabolic reprogramming. It’s clear that these updated experimental or clinical studies of PSCs may provide a promising approach for PDAC treatment in the near future.

## Background

Pancreatic ductal adenocarcinoma (PDAC) is an aggressive cancer, which is characterized by rapid progression, early metastasis, high recurrence, poor prognosis and limited responsiveness to conventional therapies [1, 2]. Worldwide, PDAC is becoming increasingly common, and has a 5-year survival rate of only 7% [3]. Despite numerous methods in PDAC treatment, including new chemotherapeutic agents, emerging immunotherapy, and advanced surgical skills, the long-term survival rate has not shown significant improvement over the past decade. There are few effective therapeutics that can extend the overall survival of patients with PDAC [4].

In recent years, it’s commonly recognized that pancreatic tumor microenvironment (TME) plays a pivotal role in PDAC carcinogenesis, progression and therapeutic resistance [5]. As a key orchestrator of TME, pancreatic stellate cells (PSCs) aroused considerable attention for its potential value in PDAC therapeutics [6]. In TME, the dynamic interactions between cancer cells and PSCs critically manipulate PDAC behaviors via diverse mechanisms.

The field of PSCs research emerged at the end of 20th century. It has been well established that PSCs are responsible for producing the desmoplastic reaction (fibrosis) of PDAC [6–8]. In addition, with exponentially increasing experimental data, more details regarding biology and functions of PSCs are coming to light [8, 9]. In particular, recent evidence indicated that PSCs exert multiple functions in paracrine actions, metabolic rewiring and intricate immune responses in PDAC.

Undoubtedly, further explorations on molecular regulatory mechanisms, PSCs-cancer cells interactions, and the clinical value of PSCs may benefit patients with PDAC. Targeting PSCs within the TME as a means of inhibiting PDAC progression is an attractive idea, which may revolutionize PDAC patient treatment and outcome [10].

### Phenotypic and functional transition of PSCs

Pancreatic stellate cells (PSCs), a periacinar star-shaped stromal cell type in healthy pancreas, were successfully isolated and cultured in 1998 [11, 12]. PSCs share many characteristics with hepatic stellate cells, including morphology, storage of lipid droplets rich in vitamin A, locations, and marker protein expressions [13, 14]. Under homeostatic conditions, quiescent pancreatic stellate cells (qPSCs) localize in basolateral aspects of acinar cells, or surround perivascular and periductal regions. qPSCs are capable of expressing several protein markers, such as glial fibrillary acidic protein (GFAP), synemin, and desmin, most of which are not specific [15, 16]. Even though the physiological roles of qPSCs haven’t been fully delineated, some functions are postulated and widely recognized. These functions include retinoid storage, basic exo-/endocrine secretion, maintenance of pancreatic tissue architecture, stimulation of amylase secretion, phagocytosis, and immunity [16] (Table 1). In general, qPSCs are believed to contribute to the normal status of the pancreas [15, 16].

**Table 1:** Biological comparison of quiescent PSCs (qPSCs) and activated PSCs (aPSCs)

	Biological behaviors or functions	Specific/selective biomarkers
qPSCs	-Store retinoids in droplets [13, 17] -Function as an immune, progenitor and intermediary cell [16] -Stimulate amylase secretion, phagocytosis and immunity [16] -Secrete MMPs and TIMPs to maintain ECM turnover; prevent collagens deposition [17] -Produce cytokines, growth factors; basic exo/endocrine secretions in a proper way [18] -May contribute to acinar regeneration [18] -Involve in maintenance of pancreatic tissue architecture [16, 18] -Help to sustain homeostasis in pancreas microenvironment [16]	desmin [15, 16] nestin [15, 16] vimentin [18] synemin [15, 16] GFAP [15, 16] NGF [15, 16]
aPSCs	-Induce desmoplastic reactions in TME [19], elevate interstitial pressure [22] -Induce hypovascularity and produce excess ECM [19, 23] -Contribute to hypoxic and low-nutrient conditions [77, 79] -Lose vitamin A lipid vacuoles [18] -Develop spindle-shaped morphology [17, 18] -Generate growth factors (GFs), cytokines, exosomes, micRNAs that enhance tumor survival, proliferation, migration and metastasis [34, 73, 84, 85] -Promote angiogenesis, PNI and EMT [44, 45, 54, 61] -Mediate chemoresistance and radioresistance [70, 105] -Contribute to complex metabolic networks in TME [112, 115] -Interact with PDA cells or other stromal components [47] -Contribute to immunosuppressive regulations and immune evasion [130–133]	α-SMA [17, 18] FAP-α [19] FSP-1 [17–19] Fibrinogen [18, 19]

Notes: Biological behaviors and functions dramatically change during phenotypic transition of PSCs. Biomarkers of qPSCs are not specific.

Abbreviations: *aPSCs* activated pancreatic stellate cells, *qPSCs* quiescent pancreatic stellate cells, *GFs* growth factors, *PNI* perineural invasion, *EMT* Epithelial-Mesenchymal Transition, *TME* tumor microenvironment, *GFAP* glial fibrillary acidic protein, *α-SMA* α-smooth muscle actin, *NGF* nerve growth factor, *FAP-α* fibroblast activation protein-α, *FSP-1* fibroblast-specific protein-1

During PDAC, resident qPSCs are activated by some risk factors (e.g. ethanol and its metabolites, chronic inflammation, smoking), environmental stress (e.g. hypoperfusion, hypoxia, oxidative stress), cellular factors (e.g. IL-1, IL-6, HIF1α, TGF-β, CCN2) and molecular regulating pathways (e.g. Wnt/β-catenin signaling, PI3K pathway), and then transform into an activated myofibroblast-like phenotype [17–19]. Activated PSCs (aPSCs) lose cytoplastic lipid droplets, and express fibroblast activation proteins, such as α-smooth muscle actin (α-SMA), and fibroblast activation protein-α (FAP-α), which serve as biomarkers for aPSCs identification and are negative prognostic factors in PDAC [17–19]. Meanwhile, aPSCs are the most important cellular source of cancer-associated fibroblasts (CAFs). As a key component in PDAC stroma, CAFs have high-level heterogeneity, the distinct subpopulations show complicated effects on growth and progression of PDAC [20, 21]. Moreover, it’s been verified that CAFs derive from diverse cellular origins, including bone marrow-derived cells (BMDCs), epithelium, and resident fibroblasts. Actually, CAFs and aPSCs are different stromal cell populations in PDAC. Even though both of CAFs and aPSCs share some common markers, none of these biomarkers are specific [20, 21]. The differences between the CAFs and aPSCs are still under debate.

**Fig. 1:**
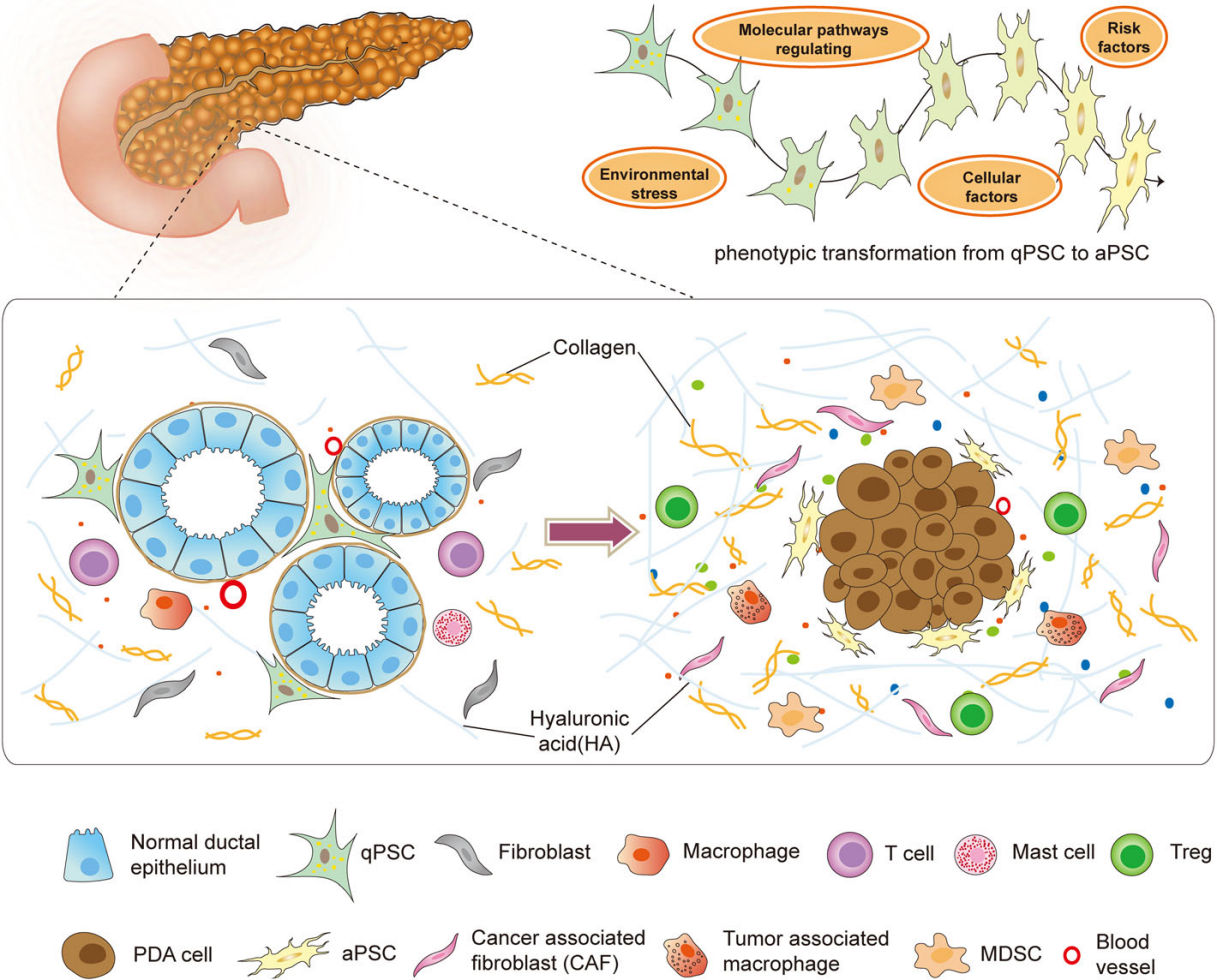
Phenotypic transition of PSCs and desmoplastic TME. qPSCs are activated by risk factors, local environmental stress, cellular and molecular regulations. During the oncogenesis, aPSCs largely contribute to fibrotic microenvironment, which is a major characteristic of PDAC. The desmoplastic TME consists of epithelial PDA cells and numerous stromal components, such as immunosuppressive cells, aPSCs, collagens and so on

aPSCs also acquire proliferative capacity, and induce desmoplastic response via synthesizing abundant extracellular matrix (ECM) [19, 22, 23]. The desmoplastic reaction is widely regarded as a hallmark of PDAC, more importantly, it’s shown to be predominantly responsible for intercellular signaling and TME reprogramming [23] (Fig. 1). However, the contribution of TME-associated desmoplasia to PDAC growth and progression is still obscure and controversial. The ‘stiff’ stroma impairs the drug delivery, some investigations indicated that depletion of tumor-associated stroma in mouse PDAC models by using enzymatic degradation of hyaluronic acid (HA) or Sonic Hedgehog inhibitor IPI926 could suppress PDAC progression [24, 25]. Oppositely, some new preclinical and clinical data suggested that stromal desmoplasia acts to restrain, rather than support PDAC progression [26]. Depletion of myofibroblast and collagen in PDAC displays immunosuppression, enhanced tumor hypoxia, EMT program and cancer stem cell-like phenotype [27]. Activation of Rho-associated protein kinase2 (ROCK2) signaling can promote PDA cells proliferation and invasiveness via matrix metalloproteinases (MMPs) release and collagen degradation [28]. Clinically, high stromal density in resected PDAC was found to be significantly associated with longer disease-free [29]. Taken together, the TME-associated desmoplasia, representing aPSCs activity, plays a dual role in PDAC. Further exploration of desmoplastic reaction is really necessary.

Additionally, persistent PSCs activation results in dramatically increased secretion of a wide variety of cytokines, chemokines, growth factors (GFs), and exosomes, which perform various pathological functions of PDAC. aPSCs-derived insulin-like growth factor 1 (IGF1), vascular endothelial growth factor (VEGF) and platelet-derived growth factor (PDGF) may promote angiogenesis, epithelial cancer cells proliferation and migration [16, 30, 31]. The overproduced matrix such as collagens, hyaluronic acid (HA) and unbalanced expression of matrix metalloproteinase and its inhibitors (MMPs, TIMPs), cause sustained fibrosis and create a physical barrier to nutrients or therapies [32, 33]. Recently, more studies suggested that aPSCs play a reciprocal role in the stroma-cancer cells interactions, which support PDAC malignant behaviors via inducing drug resistance, metabolic rewiring, and immune evasion [33, 34].

Collectively, in contrast to qPSCs, aPSCs are morphologically and functionally transformed. The activated phenotype can accelerate TME formation, and frequently promote PDAC progression through diverse pathways [35] (Table 1).

### PSCs related diverse paracrine and molecular pathways that influence invasion, metastasis, and therapeutic resistance of PDAC

PSCs are an important source of secretions in TME [7, 36]. A large number of GFs, chemokines, cytokines, miRNAs, exosomes and other soluble substances secreted by PSCs, act in an autocrine or paracrine manner to orchestrate continued PSCs activation and signaling transfer between stroma and epithelial cancer cells [37–39].

**(1) IL-6/JAK/STAT signaling:** The presence of chronic inflammation is a hallmark of PDAC progression [40]. Recent evidence implicated that PSCs is a main source for large amounts of inflammatory signals. Interleukin-6 (IL-6), a cytokine that is produced in abundance by components of stroma including PSCs and tumor-associated myeloid cells [41–43], can exert versatile functions to promote PDAC progression. In particular, IL-6 in TME can activate downstream JAK/STAT signaling via the transmembrane receptor gp130. It’s evidenced that IL-6/JAK/STAT signaling axis in TME plays an important role in the transformation from pancreatic intraepithelial neoplasia (PanIN) to carcinoma [44, 45], PDAC aggression, TME remodeling and therapeutic resistance [41, 46]. IL-6/STAT axis activated by aPSCs significantly upregulates some genes expression in PDA cells, such as major EMT regulator *Snail*, mesenchymal marker *CDH2*, and invasion related genes *CCL20*, *CFB*, *LCN2* etc, which correlates PDAC migration and evolution [42, 47]. Suppressor of cytokine signaling 3 (SOCS3) serves as a potently negative regulator that inhibits PDA cells migration, invasion, and enhances PDA cells apoptosis. Recent study demonstrated that IL-6/STAT3 axis could induce SOCS3 methylation via DNMT1, which leads to PDAC growth and metastasis [48]. Moreover, PSCs-secreted IL-6 could not only induce PDA cells proliferation via nuclear factor erythroid 2 (Nrf2)-mediated metabolic reprogramming and reactive oxygen species (ROS) detoxification [48], IL-6/STAT/Nrf2 pathway was also implied to promote EMT in PDAC [47].

Additionally, recent findings suggested the IL-6/JAK/STAT3 axis promotes the recruitment of immunosuppressive cells (e.g. MDSCs, Tregs), which hampers immune responses of PDAC [49].

In general, paracrine IL-6/JAK/STAT signaling plays a pivotal role in PSCs-PDA cells interaction and PDAC progression. Pharmacologic blockade of IL-6/JAK/STAT signaling may be a new therapeutic strategy for patients with PDAC.

**(2) Paracrine Sonic Hedgehog (SHH) signaling:** Current study shows paracrine sonic hedgehog (SHH) signaling, which involves both epithelial cancer cells and stromal cells , promotes cancer cells-stroma interactions and ultimately contributes to PDAC progression [50]. To date, it’s clear that paracrine SHH protein, which is secreted by PDA cells, serves as a hedgehog (HH) pathway ligand. SHH signaling is mediated by HH ligand binding to the membrane-localized receptor patched (PTCH) on PSCs, which relieves the inhibitory effect on a Smoothened (SMO) receptor. Derepressed SMO then leads to a cascade of cytoplasmic events in PSCs that facilitates the activation of GLI family zinc finger transcription factors, modulating targeted genes expression and eventually resulting in PSCs activation [51–55]. In turn, it’s increasingly apparent that aPSCs regulate TME remodeling and promote malignant behaviors of PDAC, including driving desmoplastic stroma [52], increased vascularity [26], uncontrolled proliferation [53, 55], perineural invasion (PNI) [54], and drug resistance [53–55]. Despite the prevailing notion, that hedgehog-driven stroma plays a critical role in neoplastic growth and PDAC progression. Inhibition of SHH signaling seems to enhance delivery of chemotherapy and improve the outcomes of PDAC [24]. However, some current data provided more uncertainties of this opinion, and even shifted to the opposite paradigm that SHH signaling may partially act to restrain PDAC growth [26]. The studies demonstrated that in spite of the success of IPI926 in treating PDAC mouse models, treatment with SMO inhibitors alone in PDAC trials showed poor clinical performance [24]. In contrast, the administration of vascular endothelial growth factor receptor (VEGFR) blocking antibody selectively improved survival of SHH-deficient PDAC, which suggested that SHH signaling-driven stroma may suppress PDAC growth partly by restraining tumor angiogenesis [26]. Generally speaking, SHH signaling exerts complicated functions in PDAC. More details need to be elucidated in the future.

**(3) Vitamin D Receptor (VDR) pathway:** Wide prospective studies have demonstrated that there is a defined inverse correlation between circulating levels of Vitamin D and risk of developing PDAC or other malignancies [56]. aPSCs express high levels of the VDR. As a notably alternative pathway, Vitamin D Receptor (VDR) signaling plays a critical role in driving conversion of qPSCs to their activated state, which then results in stromal remodeling of PDAC [57, 58]. Further investigations shows after treated with Vitamin D analogue Paricalcitol, PSCs activation can be partly reverted [16, 58]. It reveals that PSCs related VDR pathway may serve as a promising molecular target in PDAC therapy [56–58].

**(4) CXCL12/CXCR4 signaling axis:** PSCs is a predominant source of C-X-C motif chemokine 12 (CXCL12) in TME [59]. High-mobility group box 1 (HMGB1) secreted by stressed PDA cells can capture CXCL12 and then form a heterocomplex. It’s evidenced that HMGB1-CXCL12 complex interacts with C-X-C chemokine receptor type 4 (CXCR4), which is highly expressed in PDA cells under hypoxic conditions (or HIF-1α expressed strongly) [59]. The HMGB1-CXCL12 complex can induce a range of downstream aggressive behaviors, including: (1) gemcitabine treatment resistance [60]; (2) promoting proliferation, EMT, angiogenesis and metastasis of PDA cells [61]; (3) elevating MMP2,9 expression, cancer cells invasion and migration [60–62]; (4) activating other pathways, such as PI3K/Akt signaling, Ras/ERK pathway [63]; and (5) blunting immunotherapeutic efficacy, inducing immunosuppressive status [64].

**(5) Other representative paracrine signaling pathways:** Apart from mentioned above, various paracrine components or intercellular signaling are involved in PSCs activation and PSCs (or CAFs)-cancer cells interactions (Table 2), including Ca^2+^ signaling, VEGF, PDGF, Toll-like receptors (TLRs) signaling, HIF-1α signaling, TGF-β/Smad pathways, tumor necrosis factor-α (TNF-α), monocyte chemoattractant protein-1 (MCP-1), and periostin, which exert various influences on PDAC pathology [15, 34, 65–83] (Table 2). Additionally, the involvements of miRNAs and exosomes have recently being reported [84]. For example, PSCs-induced miR-210, miR-199 upregulation plays an important role in PDA cells EMT and migration [85–87], and PSCs-derived exosomal miR-21 and CCN2 partially drive PSCs fibrotic signaling [73]. However, further relevant mechanisms still need to be uncovered.

**Table 2:** PSCs mainly involved paracrine pathways and their functions

Paracrine signaling	Mediator(s)	Description	Functional roles
Toll-like receptor (TLR) signaling	DAMPs in TME	TLR9 is activated both in PDA cells and PSCs	·Pro-inflammatory effects [82] ·Up-regulated expression of PSCs-derived cytokines (e.g. CCL3, CCL11) [83] ·Recruitment of T_reg_ cells in PDAC [83]
IL-6/JAK/STAT signaling	IL-6	A versatile pathway in PSCs-PDA cells interactions	·Inducing chemoresistance, fibrotic reaction [44, 46] ·Invasive TME remodeling [47] ·Affecting other cytokines production ·Recruitment of immunosuppressive cells [49] ·Enhancing tumor aggressiveness via PSCs-PDA cells crosstalk [44, 47]
Shh signaling	SHH protein	An altered signaling between PSCs and tumor cells	·Sustaining PSCs activation and proliferation [51–55] ·Promoting vasculature and desmoplasia [52] ·Driving perineural invasion (PNI) and drug resistance [53–55] ·Tumor proliferation and progression [51, 53]
CXCL12 (SDF-1)/CXCR4 signaling	PSCs-derived SDF-1 (CXCL12)	It’s highly activated in PDAC, the elevated level is correlated with poor clinical outcomes	·Causing low response to gemcitabine treatment [60] ·Promoting PDAC progression via enhanced proliferation, EMT, angiogenesis and metastasis [61] ·Inducing over-expressed MMPs, up-regulated invasiveness and migration of tumor cells [60, 62] ·Supporting immunosuppressive environment [64] ·A potential target for PDAC immunotherapy combined with CTLA-4 or PD-L1 checkpoint block [64]
MCP-1/CCR2 pathway	MCP-1 expressed in PSCs	An important cytokine signaling mediating PSCs activation and fibrogenic ECM	·Serving as a novel component in PSC inflammatory and fibrogenic signaling [81] ·Mediating monocytes migration into pancreases and then differentiation into PSCs [81] ·Maintaining activated status of PSCs through autocrine manner [15]
Ets-2-dependent regulation	E26 oncogene homolog 2 (Ets-2) originated in PSCs	New functions unlocked about Est-2 signaling in TME of PDAC	·Stromal Ets-2 regulates chemokines production and immune cells recruitment during PDAC ·Fibroblast Ets-2 deletion leads to an increased CD8+T-cell population, and decreased presence of regulatory T cells (Tregs), MDSCs [74]
Peroxisome proliferator activated receptor-γ signaling (PPAR-γ)	PPAR-γ ligands	A nuclear hormone receptor that is characterized as the master regulator for adiopogenic properties in PSCs	·Maintenance of quiescent status of PSCs [15, 65] ·PPAR-γ ligand may enhance the phagocytic activity of PSCs, which is partially responsible for the inhibition of fibrogenesis [66]
Periostin pathway	periostin	A secretory protein mainly from PSCs, whose expression regulates behaviors of both PSCs and TME	·Periostin secreted by PSCs creates a tumor-supportive microenvironment [67] ·PSCs remains via periostin autocrine loop [68] ·Biphasic effects on PDAC development: low concentration of periostin (to 150 ng/ml) drives mesenchymal-to-epithelial phenotypical transition while high concentration (1μg/ml) promoting cancer cell migration via Akt/PKB signaling pathway [69]
microRNAs (miRNAs) and exosomes	Various miRNAs and exosomes derived from PDA cells or PSCs	A recent hot spot, covering many aspects of TME remodeling, PSCs-tumor cells interactions	·Controlling myofibroblast phenotype of PSCs [84] ·Promoting migration and proliferation of tumor cells [85, 87] ·Mediating metabolic reprogramming, TME remodeling and intracellular interplay [86] ·Delivering nutrients for cancer cells [84]
integrin	kindlin-2	Newly identified signaling	·Binding of kindlin-2 and integrin, promotes cytokines production in PSCs and further accelerating progression of pancreatic cancer [39]
galectin-1	β-galactoside-binding protein expressed in activated PSCs	A heterotrimer protein strongly expressed in the stroma of PDAC	·Promoting proliferation and chemokine synthesis of activated PSCs [70] ·Contributing to the immune escape by enhanced apoptosis and anergy of T cells [71, 72] ·Inducing SDF-1 secreted from PSCs; promoting PDAC metastasis [71, 72]
Vitamin D Receptor (VDR) pathway	Circulating Vitamin D	A promising target for PDAC treatment	·Mediating PSCs phenotypical switch and stromal remodeling [58] ·Enhancing PDAC treatment [58, 108]
Growth factors	hepatocyte growth factor (HGF), Connective tissue growth factor (CCN2), others	“Multifunctional messengers” among all components in TME	·Promoting growth, invasion, migration, and chemotherapy resistance of PDA cells [34] ·Modulator of metabolic crosstalk between tumor cells and stromal components [73] ·PSCs fibrogenic signaling [73]
Other signaling pathways	HIF-1α, ROS, NF-κB, TGF-β/Smad, VEGF, PDGF, GM-CSF and so on	Commonly present in numerous malignancies	·TME remodeling; promoting proliferation, invasion, migration, chemotherapy resistance, angiogenesis, immune evasion and other behaviors of PDA cells [75–80]

Notes: PSCs-related paracrine signaling pathways have been depicted above, including their biological roles, functional molecules and influences on PDAC behaviors

### New perspectives on PSCs-mediated molecular mechanisms that contribute to metastasis and chemoresistance of PDAC

**Fig. 2:**
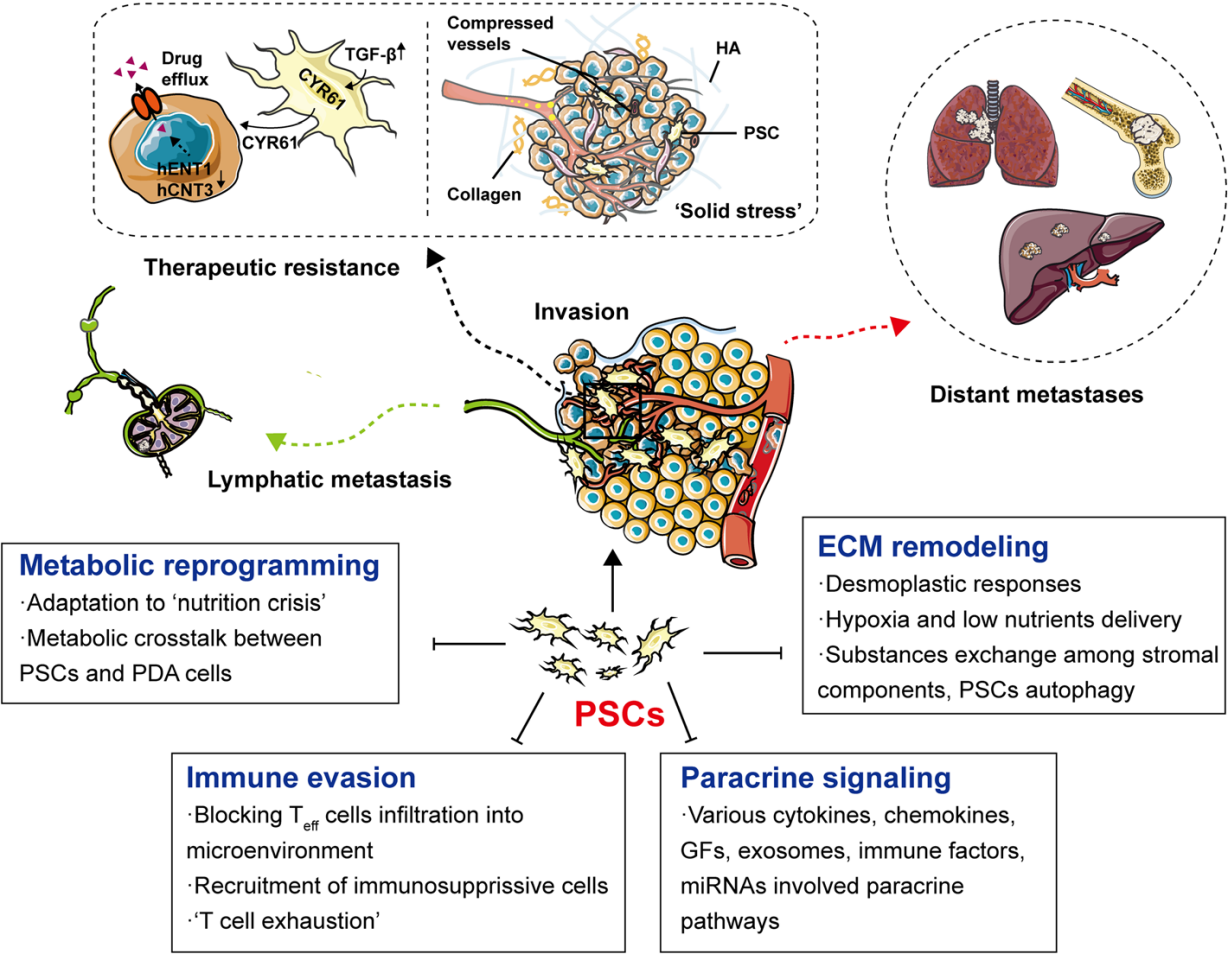
PSCs mediate invasion, metastasis, therapeutic resistance of PDAC. Multiple factors are involved in, such as immune evasion, metabolic reprogramming, ECM remodeling, various paracrine signaling and so forth

During dissemination from a primary tumor, TME plays a critical role in determining PDAC invasion and metastasis, regardless of “collective migration” or protease-dependent/independent single tumor cell invasion [88]. In particular, the plasticity of PDA cells invasion is further affected by interactions within the tumor stroma, where neighboring non-tumor cells contribute to regulating invasion or distant metastasis by a variety of mechanisms. Another troublesome problem is therapeutic resistance, which is a major contributor to the poor clinical outcomes of PDAC [89]. PSCs can exert multiple functions that are responsible for PDAC invasion, metastasis and drug resistance, such as ECM remodeling, paracrine signaling circuits, immune regulation, metabolic alterations, local proteolytic degradation, and angiogenesis [8, 40]. The updated studies focused on the PSCs’ new contributions to the biological behaviors of PDAC are as following (Fig. 2):

**(1) ‘Solid stress’ and therapeutic resistance:** Elevated tissue stiffening has become a widely accepted and passionately studied biomechanical property of fibrogenic tumors [90]. The item ‘solid stress’ refers to the physical forces caused by solid and elastic elements of the extracellular matrix and cells [90, 91]. Recently, increasing evidence suggested that the existence of solid stress is strongly linked to several hallmarks of tumor, such as proliferation, metabolism and metastasis [91–93]. In PDAC, tumor interstitial matrix (e.g. collagen, HA) and related stromal cells, such as CAFs and PSCs, mainly contribute to the solid stress. The increased solid stress is largely responsible for intratumoral vessels compression, lower perfusion and local hypoxia [24, 94]. More importantly, growth-induced solid stress tightly correlates with PDAC therapeutic resistance, and strategies designed to alleviate solid stress have the potential to improve PDAC treatment [94, 95]. It’s implied that fibrotic and hypovascular stroma reduce drugs delivery via collapsed intratumoral blood vessels, and high interstitial fluid pressure (IFP) [96, 97](Fig. 2). Continuous activation of PSCs (or CAFs) produce various ECM proteins such as HIF-1α, collagen1 (COL1), SHH and PSCs-derived periostin, which promote tumor progression, drug irresponsiveness, and contributes to tumor-supportive microenvironment and radio-/chemoresistance [24, 67, 98, 99]. Moreover, a recent study indicated that the amplified crosstalk among cancer-associated adipocytes (CAAs), tumor-associated neutrophils (TANs), and PSCs that occurs in PDAC related obesity leads to an aggravation of solid stress, increased tumor progression and reduced chemotherapy response [95]. CAAs generates an inflammatory and fibrotic TME of PDAC. Abundant adipocyte-secreted interlukin-1β (IL-1β) mediates obesity-induced TANs infiltration and PSCs activation in PDAC. Interactions between PSCs and TANs exacerbates desmoplasia in PDAC via angiotensin-II type-1 receptor (AT1) signaling, which largely hinders the delivery and efficacy of chemotherapy [95].

Apart from the theory of solid stress, another novel mechanism of PSCs-mediated therapeutic resistance has been recognizing. Transforming growth factor-β (TGF-β) mediates PSCs-expressed cysteine-rich angiogenic inducer61 (CYR61), a matricellular protein that negatively regulates nucleoside transporters hENT1 and hCNT3, which are responsible for cellular uptake of gemcitabine, largely reducing chemotherapy responses [100] (Fig. 2).

**(2) Newly identified PSCs-expressed miRNAs and PDAC progression:** miRNAs have become a hot spot for cancer research [101]. In PDAC, some new investigations highlighted the roles of PSC-expressing miRNAs in controlling the myofibroblast phenotype of PSCs and their influences on migration and proliferation of tumor cells. For example, miR-210 was reported to regulate the interactions between PSCs and PDA cells via ERK and Akt pathways [85]. Moreover, recent studies validated that miR-199a and miR-214 are upregulated in patient-derived pancreatic PSCs, and targeting them caused the dedifferentiation of aPSCs and inhibited tumor-promoting paracrine effects [86].

**(3) Autophagy in aPSCs: A potential target for PDAC:** Besides revealed involvement of PSCs in tumor invasion and metastasis (e.g. MMPs activities, EMT, angiogenesis, signaling pathways and so on), new in vivo findings suggested that autophagy in PSCs, which is induced by environmental stress and PDA cells-stroma interactions, is strongly associated with tumor T category histologic grade, peritoneal dissemination, perivascular invasion and lymph node metastasis [102]. This novel discovery might be a goal of therapeutic interest, and predict the hypothesis that targeting autophagy could be a promising candidate for treatment strategies in PDAC. Coenzyme Q10 (CoQ10), commonly known as ubiquinone, has been suggested to inhibit the activation of aPSCs by suppressing the autophagy through activating PI3K/AKT/mTOR signaling pathway [103], which may be explored to treat PSC-related pathologies and to develop anti-fibrotic approaches. Another therapeutic agent, Tocotrienols, selectively trigger aPSCs apoptosis and autophagic death by targeting the mitochondrial permeability transition pore [104]. It also unveils another potential approach to ameliorate the fibrogenesis in PDAC.

### aPSCs in PDAC metabolic reprogramming

**Fig. 3:**
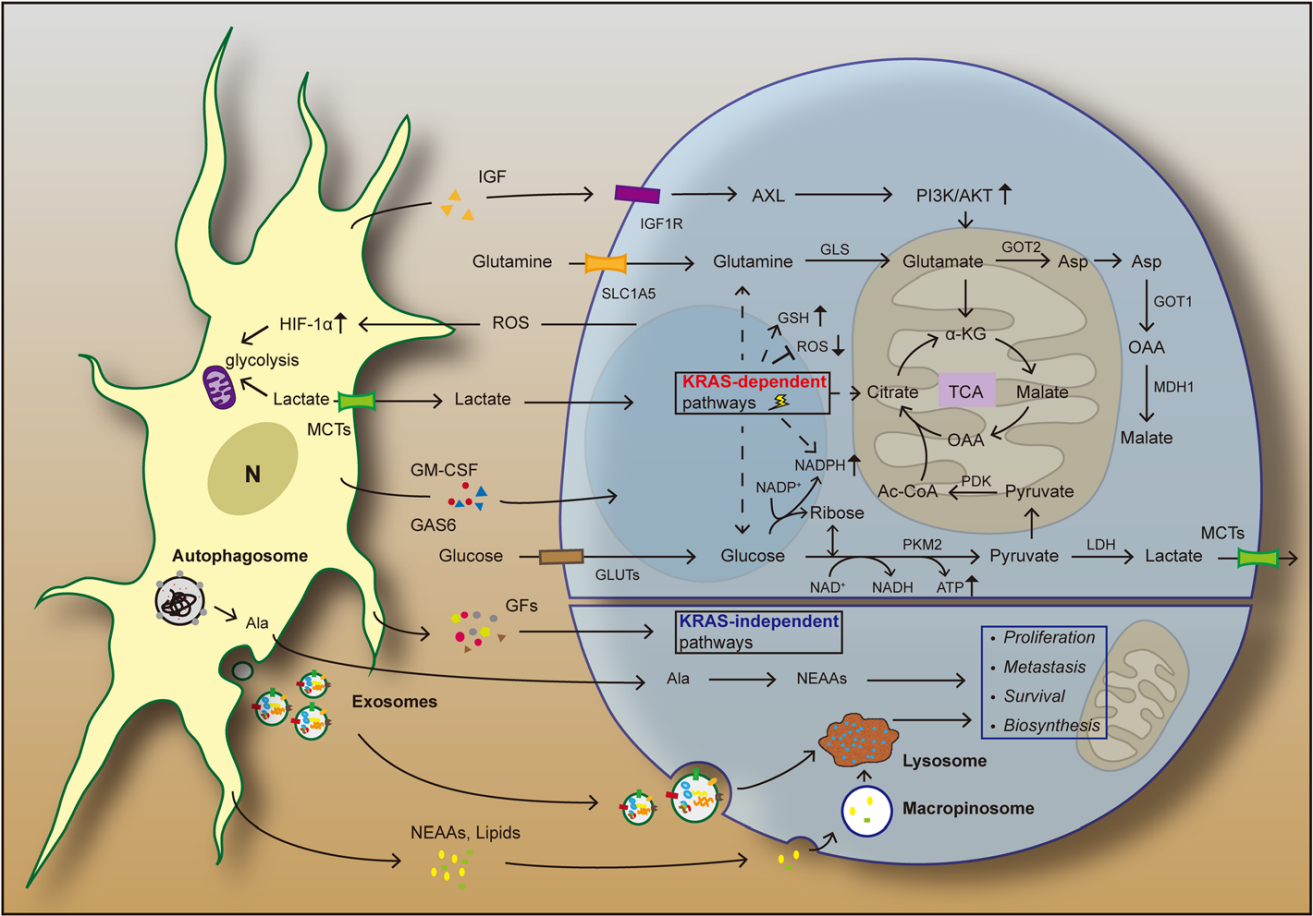
PSCs in metabolic reprogramming. In *KRAS*-dependent pathways, diverse cytokines and signaling pathways mediate metabolic interactions between PSCs and PDA cells. *KRAS*-driven glutamine (Gln) metabolism becomes a major carbon source for tumor cells survival; PSCs-derived IGF elevates mitochondrial respiration in PDA cells via IGF1R/AXL axis; *KRAS*-mutant PDA cells can obtain extracellular proteins for supporting growth through upregulated macropinocytosis. PSCs-secreted non-essential amino acids (NEAAs), such as autophagy-induced Ala, can serve as an alternative energy source to fuel PDA cells. In *KRAS*-independent pathways, PSCs-derived growth factors (GFs) and exosomes play a pivotal role in mediating survival, proliferation, metastasis, biosynthesis of tumor cells

The TME of PDAC is the major source of both interstitial pressure and oxidative stress [105]. High concentration of the PSCs-derived matrix including hyaluronic acid (HA), collagen and glycosaminoglycan, contributes to the dense fibrotic stroma and subsequently leads to intense pressure in TME [106]. As a result, elevated stromal pressure causes vascular collapse, tumor tissue hypo-perfusion, and a lack of oxygen and nutrient delivery [105, 107, 108]. The environmental stress imposes “energy crisis” to cancer cells. Despite diverse mechanisms promoting extracellular glucose acquisition via the Warburg or the reverse Warburg effect in cancer cells (e.g. HIF-1α signaling, over-expression of aerobic glycolytic enzymes like NF-kB, MCTs, PKM1/2) [108–111], obviously, enhanced glucose metabolism cannot compensate for energetic and biosynthetic shortfall completely. To sustain macromolecular biosynthesis and better tumor survival, metabolic rewiring among cancer cells and stromal components enables access to recycling nutritional substrates and alternative fuel sources for growth [112, 113]. More importantly, accumulating studies suggest that PSCs can strikingly reprogram metabolic machinery for PDAC, especially the metabolic crosstalk between PSCs and PDA cells, therefore facilitating PDAC progression and invasiveness under nutrient-limiting conditions [114, 115]. In general, understanding more details about metabolic reciprocation between epithelial cancer cells and aPSCs seems to be crucial. It’s recognized that besides environmental stresses, metabolic interplay between PDA cells and PSCs is the consequence of genetic mutations combined with comprehensive paracrine signaling regulations [108, 115, 116] (Fig. 3).

Multiple genes (e.g. *TP53, Myc, Smad4, CDKN2A*) can drive PDAC metabolic reprogramming to meet the demands of tumor-relevant anabolic processes under sterile conditions [108, 117]. Among these, oncogenic *KRAS* mutation signaling has been recently shown to play a predominant role in multiple aspects of PDAC metabolism, including adaptive metabolic responses and cancer cells-PSCs mutualism [115, 118]. More evidence has emerged that the *KRAS* mutation not only enhances glucose uptake, but also activates expression of several key enzymes in aerobic glycolysis (the Warburg effect). Furthermore, *KRAS*-driven glutamine (Gln) metabolism becomes a major source of carbon and nitrogen for cancer cells proliferation [119]. It’s demonstrated that oncogenic *KRAS* signaling mainly drives Gln conversion into aspartate for further energy generation and anabolism by activating the GOT2/GOT1/ME1 pathway, while at the same time, initiating glutathione (GSH) and NADPH biosynthesis, and inhibiting Nrf2/ME1/ROS activities to sustain cellular redox balance and enhance cytoprotection of cancer cells [113, 115, 120].

Recently, it’s become increasingly apparent that the tumor cell *KRAS* mutation manipulates signaling within both PDA cells and adjacent PSCs, and influences PSCs-tumoral metabolic interactions. *KRAS* rapidly promotes sonic hedgehog (SHH) secretion from PDA cells, which activates PSCs to induce widespread events such as overexpression of IGF1, GAS6, GM-CSF and other cytokines. This results in PSCs reciprocally sending a feedback signaling to PDA cells via IGF1R/AXL axis, activating downstream PI3K-Akt phosphorylation and increasing spare mitochondrial respiratory capacity in PDA cells [121], which elevates oxygen availability for PDA cells under hypoxia. Additionally, *KRAS*-mutant PDA cells upregulate macropinocytosis, an endocytosis-mediated bulk uptake, to import extracellular proteins, which is ultimately delivered to lysosomes for catabolism, fueling TCA cycle, essential amino acids recycling and supporting cell growth [122–124].

More interestingly, PSCs-derived non-essential amino acids (NEAAs) also provide nutrients to feed PDA cells. Recent studies revealed that PDA cells increase autophagy in PSCs via unclear mechanisms [125], and then mediate PSCs secreting alanine (Ala) [125, 126]. As a linkage of this cooperative metabolic relationship, PSCs-derived Ala is taken up by PDA cells and acts as an alternative carbon source to glucose/glutamine, shunts glucose to Ser/Gly biosynthesis, and supports lipid and NEAAs biosynthesis [115, 125].

In contrast to *KRAS* signaling related rewiring mentioned above, metabolic reprograming via *KRAS*-independent pathways has been identified [126]. GFs (e.g. connective tissue growth factor; CTGF and fibroblast growth factor-2; FGF2) and cytokines exchange between cancer cells and surrounding PSCs have been proved to be pivotal in metabolic crosstalk [126–128]. Furthermore, PSCs-derived exosomes contain various biomolecules, including mRNA, miRNA, intracellular metabolites (e.g. amino acids, acetate, stearate, palmitate, and lactate), which enter PDA cells, both fuel the tricarboxylic acid cycle (TCA cycle) and enhance tumor growth in a manner similar to macropinocytosis [84, 126, 128, 129].

Collectively, the description of these new metabolic crosstalk pathways further highlights that (1) PSCs play a key role in intra-tumoral metabolic networks, and (2) the PDA cells-PSCs metabolic “coupling” contributes significantly to PDAC development under nutrient-poor environment [115].

### PSCs-mediated immunosuppressive microenvironment in anticancer immunity

**Fig. 4:**
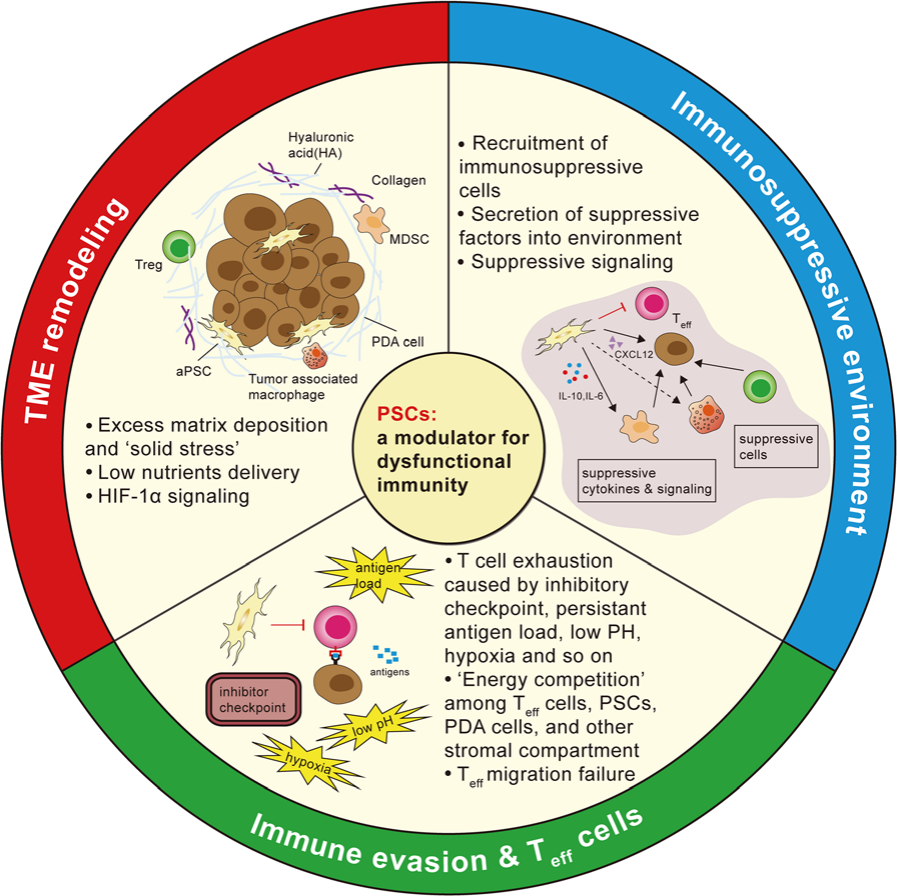
Immunosuppressive modulator role of PSCs. PSCs induce TME remodeling, dense matrix caused hypoxia and hypo-vascularity impair T cells infiltration and their nutrition obtaining; multifactorial T cell exhaustion attenuates T_eff_ functions; PSCs-derived suppressive factors (such as IL-6, CXCL12), suppressive signaling and recruitment of suppressive cells (such as MDSCs, T_reg_ cells, TAMs) create an immunosuppressive TME in PDAC

Despite continuous progress in understanding the immune-dependent regulations of PDAC and the development of immunotherapies [130], therapeutic advances have been insufficient [131, 132]. Elucidating a method to enhance antitumor immunity and immunotherapy seems to be a challenge in PDAC treatment. Immune evasion and T cell dysfunction can be mediated by a variety of mechanisms, such as the immunosuppressive microenvironment in PDAC patients, which involves interactions among tumor cells, infiltrated immune cells and stromal components [108, 117], and makes a major hurdle for immune responses [133]. As a currently compelling role, PSCs display multiple effects on immunosuppressive regulation that makes PDAC therapeutics more difficult [134, 135] (Fig. 4).

First, α-SMA^+^ or FAP^+^ PSCs play a pivotal role in TME remodeling. PSCs-mediated desmoplasia results in excess matrix deposition in TME, which has been postulated to limit T cell infiltration and function once recruited into tumor site [136, 137]. A striking cell-intrinsic pathway impacting cancer immunity is focal adhesion kinase (FAK), a tyrosine kinase that regulates T cell survival, antigen sensitivity, cytokine production and migration [138]. FAK1 level is elevated in PDA cells and correlates with robust fibrosis and poor CD8^+^ T cell accumulation. The rigid ECM components secreted by PSCs, such as collagen or fibronectin, induce Rho-associated coiled-coil kinase-dependent activation of FAK1, greatly contributing to suppressed anticancer immunity [138].

Second, desmoplastic response creates hypoxic and avascular conditions, which imposes considerable energetic constraints on tumor cells, PSCs and immune cells [115]. As we mentioned above, PSCs constitute the major source of cancer-associated fibroblasts (CAFs) in PDAC. Paracrine signaling from neighbor PSCs (or CAFs) and CAFs-tumor cells interactions lead to metabolic reprogramming, by which cancer cells express more nutrients import molecules (e.g. GLUT1, MCTs, ASCT2, LATs) to obtain fuel sources for survival [139]. Elevated indoleamine-2,3 dioxygenase 1 (IDO1) and arginase (ARG1, ARG2) in metabolically altered CAFs may deplete tryptophan and arginine, which are crucial for T effector (T_eff_) cells’ proliferation and activation [139, 140]. Meanwhile, confronted with “metabolic competition”, lack of glucose impairs T cell’s anti-tumor immunity and secretion of Interferon-γ (IFN-γ) [141, 142], while low lipid support results in TNF receptor associated factor 6 (TRAF6) deficiency, which inhibits long-lasting memory CD8^+^ T cells formation [143].

Significantly, it has been commonly assumed that T cells in the context of established progressing cancer patients exhibit an exhausted status (termed as “T cell exhaustion”) due to various factors, such as persistent tumor antigen load, inhibitory checkpoint signaling pathways (e.g. PD-1, LAG-3, CTLA-4), cell-intrinsic tolerance programs, and, more importantly, the complex immunosuppressive environment [144]. In PDAC, PSCs to a large extent mediate physiological changes in TME (e.g. hypoxia and low pH). HIF-1α activation can suppress immunity or promote tumor evasion, however the underlying molecular mechanisms remain to be further identified [145, 146].

PSCs also secrete numerous soluble cytokines that contribute to “T cell exhaustion” and dysfunction. It’s well evidenced that PSCs-derived CXCL12 (also named stromal-derived factor-1, SDF-1) can limit cytotoxic T cells trafficking, mediate macrophages’ differentiation into a pro-tumor M2 phenotype (tumor-promiting), and recruit myeloid-derived suppressor cells (MDSCs), tumor-associated neutrophils to the tumor site [147]. At the same time, CXCL12/ SDF-1 bound to PDA cells can inhibit T cell access to immune responses [148]. Recent clinical trials demonstrated that inhibiting CXCR4, a CXCL12 receptor, can dramatically promote T-cell accumulation and synergize with the checkpoint antagonist, α-PD-L1, to cause cancer regression [64, 148].

Similarly, another versatile PSCs/MDSCs derived pro-inflammatory cytokine, interleukine-6 (IL-6), can suppress cytotoxic T lymphocyte (CTL)-driven antitumor immunity by multiple mechanisms, including impairing T_eff_ cells trans-endothelial migration, activation of T_reg_ cells Foxp3+ or tumor-associated macrophages (TAMs), and inducing imbalance of T_reg_/ T_eff_ activities [149, 150]. Moreover, large amounts of PSCs-derived suppressive cytokines such as IL10, TGF-β, VEGF, MCP-1, GM-CSF, PGE_2_, also contribute largely to immune evasion and anti-tumor hyporesponsiveness of PDAC [144, 151].

In short, with regard to the immunity regulation in PDAC, PSCs seem to be a powerful immunosuppressive modulator via numerous pathways. Targeting PSCs may pave a novel avenue for enhancing immunotherapies for PDAC.

## Conclusions

PSCs surrounding tumor cells is an emerging stromal component that has been receiving huge attention recently. As a powerful tumor contributor, there is accumulating evidence supporting the multiple roles of PSCs in the establishment of TME, such as regulating environmental homeostasis and metabolic reprogramming, supporting tumor survival, immune evasion, invasion, distant metastasis and therapeutic resistance. The interplay between cancer cells and PSCs is increasingly recognized as a main driver for PDAC progression. Although development on basic studies and therapeutic strategies targeting PSCs have been revealing, more details on PSCs and PDAC treatment remains to be illustrated. It’s promising that further understanding about PSCs will open new avenues for translational medicine and more meaningful clinical therapies for PDAC.

## Abbreviations

Ala: Alanine
aPSCs: activated pancreatic stellate cells
ARG: Arginase
AT1: Angiotensin-II type-1 receptor
BMDCs: Bone marrow-derived cells
CAAs: Cancer-associated adipocytes
CAFs: Cancer-associated fibroblasts
COL1: Collagen 1
CoQ10: Coenzyme Q10
CTGF: Connective tissue growth factor
CTL: Cytotoxic T lymphocyte
CTLA-4: Cytotoxic T lymphocyte-associated antigen-4
CXCL12: C-X-C motif chemokine 12
CXCR4: C-X-C chemokine receptor type 4
CYR61: Cysteine-rich angiogenic inducer61
ECM: Extracellular matrix
ERK: Extracellular signal-regulated kinase
FAK: Focal adhesion kinase
FAP-α: Fibroblast activation protein-α
FGF2: Fibroblast growth factor-2
FSP-1: Fibroblast-specific protein-1
GFAP: Glial fibrillary acidic protein
GFs: Growth factors
Gln: Glutamine
Gly: Glycine
GOT1: Aspartate aminotransferase 1
GOT2: Aspartate aminotransferase 2
GSH: Glutathione
HA: Hyaluronic acid
HH: Hedgehog
HIF-1α: Hypoxia inducible factor 1α
HMGB1: High-mobility group box 1
IDO1: Indoleamine-2,3 dioxygenase 1
IFN-γ: Interferon-γ
IFP: Interstitial fluid pressure
IGF1: Insulin-like growth factor 1
IL-1: Interleukin-1
IL10: Interleukin-10
IL-6: Interleukin-6
JAK: Janus kinases
LAG-3: Lymphocyte-activation gene 3
MCP-1: Monocyte chemoattractant protein-1
MCTs: Monocarboxylate transporters
MDSCs: Myeloid-derived suppressor cells
ME1: Malic enzyme 1
miRNAs: microRNAs
MMPs: Matrix metalloproteinases
NADPH: Hicotinamide adenine dinucleotide phosphate
NEAAs: Non-essential amino acids
NF-kB: Nuclear factor-kB
Nrf2: Nuclear factor erythroid 2
PD-1: Programmed cell death protein 1
PDAC: Pancreatic ductal adenocarcinoma
PDGF: Platelet-derived growth factor
PI3K: Phosphatidylinositol 3 kinase
PKM1/2: Pyruvate kinase M 1 and 2
PNI: Perineural invasion
PSCs: Pancreatic stellate cells
PanIN: Pancreatic intraepithelial neoplasia
qPSCs: quiescent pancreatic stellate cells
ROCK2: Rho-associated protein kinase2
ROS: Reactive oxygen species
SDF-1: Stromal-derived factor-1
Ser: Serine
SHH: Sonic hedgehog
SMO: Smoothened
SOCS3: Suppressor of cytokine signaling 3
STAT: Signal transducer and activator of transcription
TANs: Tumor-associated neutrophils
T_eff_: T effector cells
TGF-β: Transforming growth factor-β
TIMPs: Tissue inhibitors of metalloproteinases
TLRs: Toll-like receptors
TME: Tumor microenvironment
TNF-α: Tumor necrosis factor-α
TRAF6: TNF receptor associated factor 6
Treg: Regulatory T cell
VDR: Vitamin D Receptor
VEGF: Vascular endothelial growth factor
VEGFR: Vascular endothelial growth factor receptor
α-SMA: α-smooth muscle actin
